# Quantum Dot Applications Using Kinetic Data: A Promising Approach for Enhanced Analytical Determinations

**DOI:** 10.3390/bios15030167

**Published:** 2025-03-05

**Authors:** Rafael C. Castro, Ricardo N. M. J. Páscoa, David S. M. Ribeiro, João L. M. Santos

**Affiliations:** LAQV/REQUIMTE—Laboratório Associado Para a Química Verde da Rede de Química e Tecnologia, Laboratory of Applied Chemistry, Department of Chemical Sciences, Faculty of Pharmacy, University of Porto, Rua de Jorge Viterbo Ferreira n° 228, 4050-313 Porto, Portugal; rafael.castro.cl@hotmail.com (R.C.C.); joaolms@ff.up.pt (J.L.M.S.)

**Keywords:** quantum dots, kinetics, second-order advantage, fluorescence, chemometrics

## Abstract

The acquisition of kinetic data in QD-based PL sensing methodologies has been revealed to be an auspicious alternative in applying these nanomaterials in analytical chemistry, enabling enhanced discrimination and quantification of analytes, even in complex sample matrices. The accessibility of kinetic measurements, which use routine laboratory instrumentation, is a significant advantage that increases the practicality of this methodology. The simple acquisition of these kinds of second-order data combined with chemometric analysis can ensure accurate results in environmental, biomedical, and food monitoring applications. These developments emphasize the vital importance of kinetic approaches in increasing sensitivity, improving analyte discrimination, and making the application of QDs in complex samples possible.

## 1. Introduction

Quantum dots (QDs) have unusual properties, making them one of the most promising nanomaterials in nanoscience and nanotechnology, with a panoply of applications, including bioimaging/biosensing [1,2,3,4], photocatalysis [5,6,7], chemical analysis [8,9,10], and electronics, especially in their use in light-emitting diodes (LEDs) or light-harvesting systems [11,12]. Additionally, QDs can be arranged in single-electron semiconductor quantum dot arrays, which can be applied in ultra-sensitive detection and quantum computing [13,14,15]. There has been a growing interest in the scientific community in applying these nanomaterials in analytical chemistry due to their exceptional optical properties. For example, the simultaneous excitation of multiple QDs with distinct sizes and/or compositions using a single excitation wavelength for multiplexed detections is possible due to their high molar attenuation coefficients and broad excitation bands [16,17,18]. Additionally, the combination of multiple fluorophores within the same probe without significant spectral overlap due to their narrow emission bands allows for obtaining enhanced detection capabilities, generating specific analyte response profiles [19]. It is also important to emphasize the possibility of obtaining nanocrystals that emit light across the entire UV/visible-to-infrared electromagnetic spectrum only by adjusting the size of these nanomaterials during the synthesis [16,17,18]. In addition, the ability to detect a multitude of analytical targets with greater selectivity is provided by the enhanced specific recognition capacity of QDs, which could be obtained because of their highly tunable surface chemistry, enabling their conjugation with numerous biomolecules and polymers, such as antibodies, oligonucleotides, proteins, and molecularly imprinted polymers (MIPs) [20,21]. Finally, QDs can provide enhanced sensitivity for sensing applications as they possess high photoluminescent (PL) quantum yield (QY) [16,17,18].

QDs have two essential characteristics that make them valuable sensing platforms for implementing kinetic assays. The first one is their resistance to photobleaching, which is much higher than that of organic fluorophores, permitting prolonged irradiation time during time-based measurements. The other one is their long PL lifetimes, ranging from tens to hundreds of nanoseconds, making them ideal nanomaterials for tracking dynamic processes over time [10].

Despite all the aforementioned advantages, broader applications in chemical analysis, particularly in samples with complex matrices, could be a significant drawback because of the QDs’ limited selectivity, which occurs when their surfaces are not functionalized with specific recognition elements [9,10]. The analysis of PL data using chemometric models has emerged as a useful strategy to circumvent this issue [8,10]. In this context, a more efficient description of the relationships between samples and analyzed variables can be obtained, which allows for a better understanding of complex systems and improves the analytical results.

Three-dimensional datasets, including Excitation–Emission Matrix (EEM) and kinetic data, also called second-order data, can be processed by appropriate chemometric models, which provide the advantage of circumventing the occurrence of unexpected sample components not considered during the calibration step (second-order advantage). This can be extremely important for distinguishing between different components or predicting potential interferences that impact analytical results, particularly when analyzing complex samples, such as food, environmental, or biological samples [8,10]. So, in these cases, acquiring more complex data such as second- or even higher-order data can successfully mitigate the effects of these interferents, which allows for obtaining more accurate and reliable analytical results as well as better discrimination in the qualitative analysis compared to what would be obtained with zeroth- or first-order data.

Despite being the most explored second-order data in QD-based methodologies, acquiring EEM data requires expensive and specific instrumentation. A simpler and alternative way to acquire second-order data involves recording over time the QD PL spectra modulation in the presence of increasing analyte concentrations, which can be easily obtained using a common fluorimeter [8]. Nevertheless, despite its simplicity, only a very restricted number of analytical methodologies have explored this possibility [8].

Kinetics is essential in chemistry, biology, and environmental science because it enables an enhanced understanding of how and why reactions occur at certain rates and under specific conditions, providing relevant information about mechanisms and dynamics [22]. Consequently, kinetic approaches are frequently employed in enzyme studies, quality control processes, environmental monitoring, and drug development [22].

Kinetic determinations in analytical chemistry involve using techniques and methods to measure the variations in the concentration of reactants or products over time and determine the rate at which a given reaction or chemical process occurs. Kinetic assays are mostly interesting for quantitative analysis as relating the reaction rate to the concentration of the analytes in a sample provides diverse advantages regarding fixed-time assays, where measurements are performed at a single time point, usually after the reaction development. On the contrary, the kinetic approach enables the continuous measurement of reaction rates, which provides valuable insights into reaction mechanisms and consequently facilitates more accurate quantification of analyte concentrations in complex samples [22,23].

Additionally, kinetic assays usually exhibit a wider dynamic range than fixed-time assays, allowing for the accurate determination of analytes across a broader concentration range. Effectively, kinetic assays can measure reaction rates at multiple time points, accommodating a wide range of analyte concentrations. In contrast, in fixed-time assays, the measurements may be limited by the linear range of detection. This wider dynamic range allows for the accurate quantification of analytes in diverse sample matrices, demonstrating the high versatility and applicability of kinetic assays.

In summary, the ability of kinetic assays to provide real-time monitoring, higher accuracy, and a wider dynamic range makes them indispensable analytical methods for quantitative analysis in the fields of analytical chemistry and biochemistry [23,24].

Kinetic determinations using quantum dots as PL nanoprobes represent an interesting challenge in analytical chemistry. By taking advantage of the abovementioned properties of QDs, researchers have opened new avenues for studying real-time reaction kinetics with improved sensitivity and accuracy. This review provides a comprehensive overview of recent advances, applications, and future perspectives of kinetic determinations using quantum dots as PL probes. Despite the potential of this symbiosis, the number of works on this topic is relatively low and can be divided into two main areas of focus: seeking selectivity enhancement or analytical performance improvement.

## 2. Kinetic Strategies in Quantum Dot Applications

### 2.1. Selectivity Enhancement by Coupling with Chemometrics

QD-based PL sensing platforms have been widely used in analytical applications; however, their poor selectivity is the largest issue that remains to be solved. Effectively, QDs lack molecular recognition abilities without appropriate surface functionalization, becoming highly reactive sensing elements because of their susceptibility to interact with various chemical species in a non-selective way. This limitation makes it difficult to detect multiple analytes in a single analysis or to monitor them in the presence of interfering substances [9,10].

The capability of QDs to serve as sensing elements is attributed to the modulation of their optical properties upon interaction with the target molecule, with the nanoparticle surface playing a crucial role [8,10,16,18]. The influence of the QDs’ surface on their optical properties can be understood in terms of trap states arising from structural defects, such as dangling bonds or adsorbates present at the surface, among others. Depending on its properties, the analyte can induce changes in the intensity of the QDs’ PL emission, either enhancing or impairing the efficiency of electron-hole recombination, leading to either a photoluminescence-enhancing or -quenching effect, respectively [9,10].

Consequently, research efforts have been focused on developing strategies to overcome this challenge. These efforts seek to enable accurately determining one or more analytes in a single analysis and monitoring analytes in complex sample matrices containing interfering species. Some strategies can be highlighted to circumvent the reduced selectivity of QDs, including the following:

(i) separation techniques to separate the analyte from the sample matrix, reducing the presence of potential interferents. However, these strategies involve labour-intensive and time-consuming procedures, which frequently require using strong acids, heating, auxiliary oxidants, and organic solvents [25]; (ii) tuning the surface chemistry of QDs through the functionalization of their surface with appropriate molecular recognition elements, namely oligonucleotides, polymers, biomolecules, or enzymes. This allows QDs to obtain specific recognition capabilities to detect target analytes. Surface functionalization is essential for multiplexed detection approaches or analyte quantification, especially in complex samples. Thus, the enhanced recognition capabilities ensure that the PL modulation of the functionalized sensing platform results specifically from the interaction with the target analyte. The success of this strategy depends on the efficiency of the recognition element, which guarantees selectivity and consequently allows multiplexed detection. However, this approach is usually elaborate, time-consuming, and expensive [10,20,26]; (iii) exploring Förster Resonance Energy Transfer (FRET) sensing schemes can help overcome selectivity issues in QD-based assays by taking advantage of the distance-dependent energy transfer process between a donor fluorophore and an acceptor. In FRET-based assays, energy is transferred from the donor to the acceptor when they are close enough (less than 10 nm), reducing the donor’s fluorescence. This process can be used successfully in the specific detection of molecular interactions or binding events due to its high sensitivity to changes in distance. This allows for specifically detecting interactions between the QDs and target analytes, minimizing interference from non-target molecules. These strategies have several drawbacks such as the need for advanced materials, complex experimental setups, sophisticated instrumentation, restrictions on the number of fluorophores that can be combined in a given experiment, and complex data interpretation procedures. Furthermore, the selectivity of FRET-based assays is still poor without efficient functionalization of surface chemistry [27,28,29]; (iv) using chemometric models to analyze and process the acquired PL data. This approach enables the identification and quantification of each analyte in complex sample matrices or mixtures, even in the presence of interferents. Chemometric models that employ statistical and mathematical methods allow for describing the relationship between PL data and analytical parameters, facilitating a deeper understanding of the dataset. Extracting more valuable insights from gathered data through chemometric analysis facilitates the selective analysis of an analyte, even without any sample purification steps or improvements in QDs’ selectivity in a FRET process or QD surface functionalization. This selectivity and the accuracy of the methodology can be further improved depending on the complexity of the acquired data and the selection of the most appropriate chemometric models. Therefore, the chemometric analysis of PL data represents a simpler and more economical strategy to overcome selectivity problems [30,31,32].

In the following sections, the use of chemometric models to circumvent selectivity issues related to QD-based sensing platforms will be discussed in detail.

### 2.2. Relating Data Complexity and Their Processing Using Chemometric Models

Chemometrics, a term first introduced by Svante Wold in the 1970s [33], refers to the science of extracting valuable chemical information from complex experimental systems and transforming the acquired data into meaningful insights [33,34]. While univariate methods analyze one variable independently of others, they fail to recognize the inter-correlation among multiple variables. In contrast, multivariate strategies can consider various variables, ensuring a more comprehensive interpretation of the available data [34].

The instrumental data obtained for QD-based PL methodologies can be categorized based on their complexity, each type offering distinct advantages. Analytical data are classified in terms of increasing complexity as zeroth-order, first-order, second-order, and higher-order data, depending on the characteristics of the instrument or method used to acquire it. It is anticipated that as the complexity of the collected data per sample analysis increases, more refined quantitative or qualitative analytical estimations can be achieved, along with greater selectivity [35,36].

**Figure 1:**
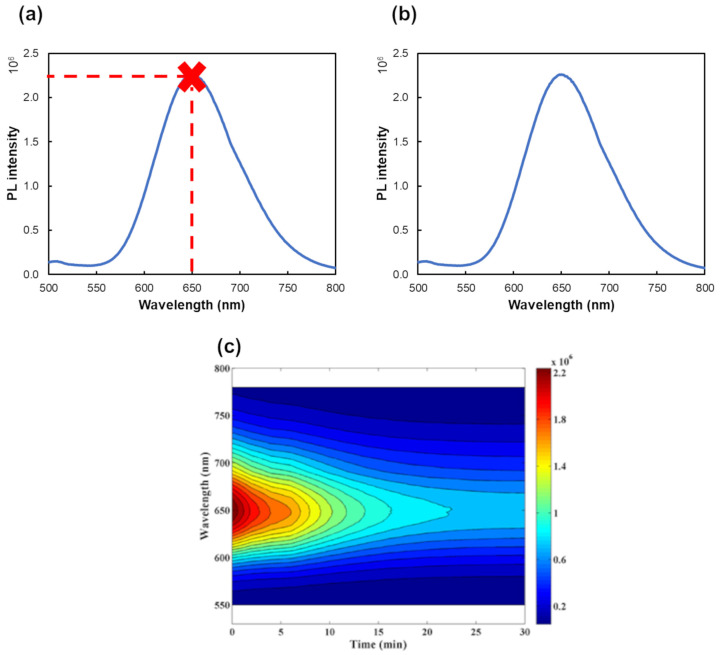
Summary of the different data structures that can be obtained for a sample using PL-based methodologies. (**a**) Zeroth-order data, representing fluorescence intensity at a single wavelength as a function of analyte concentration (red cross); (**b**) First-order data, corresponding to the fluorescence emission spectrum at a fixed excitation wavelength; and (**c**) Second-order data, related to an Excitation-emission matrix or the evolution of the sample’s PL spectrum over time at a fixed excitation wavelength. Adapted with permission from [37]. Copyright 2021 Elsevier.

Zeroth-order data are acquired when the instrument generates a single output per sample. An example is the acquisition of a fluorescence intensity, at a specific wavelength, for each analyte concentration (Figure 1a). This implies that each sample is defined by a single numerical value. In analytical contexts, it is crucial to emphasize that zeroth-order data, employed in univariate calibrations, necessitate complete selectivity towards the analyte under investigation and are applicable only to samples with a known composition and that lack interfering species [34,36].

First-order data consist of vector data for each sample, such as acquiring a PL emission spectrum at a fixed excitation wavelength (Figure 1b). This spectrum includes a series of intensity values at various wavelengths, forming a data vector. Analyzing these first-order data with appropriate first-order multivariate calibration methods enables the circumvention of selectivity limitations through the application of efficient mathematical algorithms. This is known as the first-order advantage, which allows for the quantification of the analyte in samples containing known interferents, if these interferents are included in the standard solutions used during calibration. However, unexpected constituents can make the quantification of the target analyte impossible [34,36].

In second-order data, a data matrix is obtained for each sample (Figure 1c). These complex data contain extractible complementary information, which, through second-order advantage, enables the monitoring of the analyte, even when unexpected interferences are present. This can be especially relevant in complex samples (such as biological, environmental, or food samples), where the sample composition is unknown and, therefore, unexpected interferents may be present [34,36].

**Figure 2:**
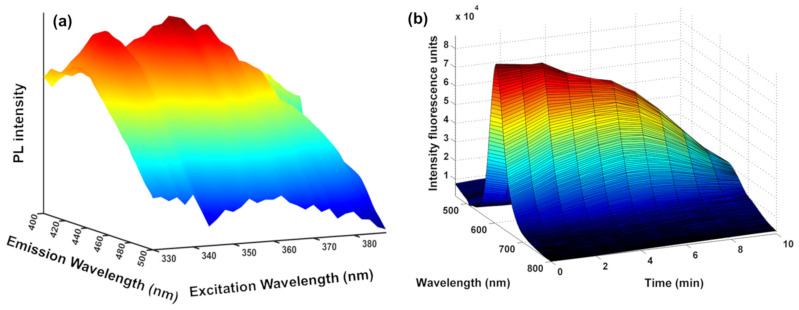
Second-order data using QD-based methodologies: (**a**) excitation–emission spectra (λ_em_ ranging from 330 nm to 380 nm and λ_ex_ ranging from 400 to 500 nm) of CDs and (**b**) PL kinetics data of the combined nanoprobe encompassing CdTe/AgInS_2_ QDs upon interaction with acetylsalicylic acid over 10 min. The colors represent the intensity of the emission, with red indicating higher intensity and blue indicating lower intensity. Adapted with permission from [38,39]. Copyright 2024 Elsevier and MDPI 2023.

Second-order data in QD-based PL analytical methodologies can be acquired by two main approaches: (i) employing a spectrofluorometer able to record an EEM (Figure 2a) or (ii) monitoring the kinetics of the QDs/sample interaction, where the evolution of the emission spectrum over time at a fixed excitation wavelength is assessed (Figure 2b). Another alternative, but unusual, strategy involves the use of hyphenated instruments with multiblock data analysis techniques. In this approach, data of diverse natures are collected from different instrumental modes, extracting complementary and useful information from the sample [8,10].

In fact, either the EEM or the kinetics of the QDs/sample interaction ensures ample spectral information essential for circumventing the presence of unexpected interfering compounds in complex samples. On one hand, the EEM can enhance the specificity of fluorescence spectroscopy. By collecting emission spectra at various excitation wavelengths, EEMs generate a matrix of PL intensities. This matrix provides a comprehensive overview of the sample’s fluorescence behavior across different excitation and emission wavelengths, allowing for a more detailed analysis and improved specificity in identifying and characterizing fluorescent compounds or substances present in the sample. On the other hand, the collection of the emission spectra at different times of the QDs/analyte interaction allows for obtaining more information and better understanding the interaction between them. However, when quantum dots (QDs) are utilized as a sensing platform, the complexity of the matrices and the data they generate requires the use of sophisticated mathematical models and computational techniques for accurate decoding. Therefore, effectively handling the complexity of data matrices in QD-based methodologies necessitates a comprehensive approach. This involves combining advanced experimental procedures with robust data analysis strategies to extract meaningful insights [8,10].

Within the two main alternatives discussed to obtain second-order data using QDs as fluorescent probes, EEM data acquisition demands costly and specialized instrumentation. Alternatively, the time-based recording of QD PL spectra modulation in the presence of increasing analyte concentrations, easily monitored using a common fluorimeter, offers a straightforward method to gather second-order data, without requiring an investment in specialized equipment. Nevertheless, a very restricted number of analytical methodologies have explored this possibility [8]. It must be considered that the utilization of kinetic data carries analytical significance only if consistent variations in the optical properties of the QDs are observed over time. However, in certain instances, when QDs are mixed with the analyte, their optical properties are swiftly altered, thereafter remaining unchanged for extended periods of time, rendering them analytically irrelevant.

There are several algorithms available for second-order data analysis, but only a few have been used in QDs applications (namely, multivariate curve resolution, unfolded partial least squares, multiway partial least squares, and artificial neural networks). These algorithms can be categorized into three different groups: noniterative (e.g., direct trilinear decomposition), iterative (e.g., parallel factor analysis and multivariate curve resolution with alternating least squares), and residual bilinearization (e.g., unfolded partial least squares and multiway partial least squares, both followed by residual bilinearization) [40]. It is beyond the scope of this manuscript to conduct an extensive and detailed analysis of all available algorithms for second-order data analysis. Instead, only a brief overview of the algorithms described in this manuscript used in QD applications will be made. For more detailed information about these algorithms, we recommend reading the reference [40]. The multivariate curve resolution with alternating least squares (MCR-ALS) extracts meaningful information from a system through bilinear model decomposition. The application of constraints (e.g., unimodality and non-negativity, among others) during ALS optimization helps reduce the problem of rotational ambiguity, thereby improving model accuracy and enabling the identification of components in the system. Additionally, applying the correlation constraint enables quantitative measurements of the components present in the system. Thus, this model allows handling second-order data that do not follow a trilinear structure, featuring overlapping signals, background contributions, and noise, while obtaining the second-order advantage [41,42]. Unfolded partial least squares (U-PLS) is capable of handling second-order data by unfolding them into vectors and performing a bilinear model decomposition [40,43]. Calibration then begins using these vectors (X data) against the concentration values of the parameters to be analyzed (Y data), aiming to explain the most variance contained in the X data and maximize its covariance with Y data. During this process, it is important to select the optimal number of latent variables to avoid overfitting. The most common method for estimating the best number of latent variables is the leave-one-out cross-validation method. After optimizing using the calibration set, the validation samples are projected to assess the accuracy of the developed model. However, in the presence of unexpected constituents in the validation samples, the U-PLS method may not be able to fully mitigate their influence, but it can identify them as outliers because their residuals will be significantly higher. Therefore, a complementary algorithm named residual bilinearization (RBL) can be employed to reduce the residual errors of these samples to values similar to the noise of the instrument used. Multiway partial least squares (N-PLS) operates under the same principles as U-PLS but without the need to unfold the second-order data and maintain covariance maximization [44]. This method is often considered superior to unfolding methods such as U-PLS due to its stable multiway decomposition, which makes it less susceptible to handling noisy signals and easier to interpret without losing information through unfolding [44]. Artificial neural network (ANN) models are capable of modeling complex nonlinear data and consist of three layers (input, hidden, and output layers), also known as architecture, which are interconnected in multiple ways with the next layer. These models can “learn by example” through iterative training processes such as backpropagation, where the weights of the neurons within each layer are adjusted to minimize the error using only the calibration set [45,46]. During this process, it is crucial to adjust several parameters properly to avoid overfitting of the model [46]. The primary difference between multilayer feed-forward (MLF) and radial basis function (RBF) NNs lies in the activation function used, where MLF typically employs sigmoid functions to introduce nonlinearity, while RBF uses radial basis functions such as the Gaussian function. After optimization, validation samples are projected to assess the accuracy of the developed ANN models [45,46].

It should be highlighted that the MCR-ALS, U-PLS/RBL, and N-PLS/RBL models are more suitable for linear data, although U-PLS/RBL and N-PLS/RBL can handle mild nonlinear data [47]. When dealing with nonlinear data, ANNs are often the best option.

### 2.3. Kinetic Determinations to Enhance Analytical Performance Using QDs as PL Nanoprobes

Acquiring the time-based evolution of QD PL spectra resulting from the interaction between QDs and the target analyte could be regarded as having great analytical value for analyte concentration quantification. Given the complex structure of second-order data, these data should be processed using suitable second-order multivariate calibration methodologies. In this way, not only is analyte quantification simplified but it also allows for mitigating the presence of uncalibrated interfering species, whose effects are thus excluded from the quantification process. However, using QD-based PL kinetics data processed by chemometric models for analytical purposes remains relatively limited among the methodologies proposed in the scientific literature.

**Figure 3:**
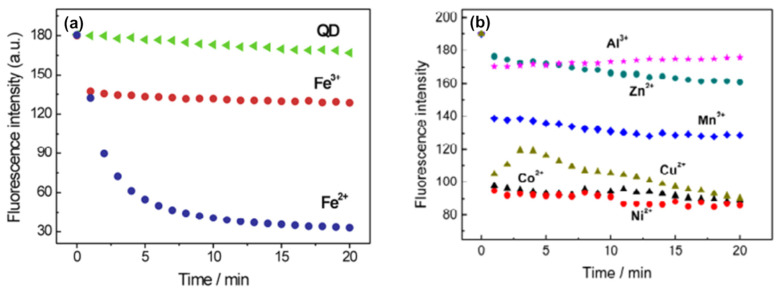
(**a**) Variation in the PL signal of GSH-CdTe QDs during 20 min without and with Fe^2+^ and Fe^3+^. (**b**) Kinetic behavior of GSH-CdTe in the presence of different transition metal ions. Adapted with permission from [48]. Copyright 2009 American Chemical Society.

In the first proposed methodology from 2009 (Table 1), which involved studying the interaction between multiple analytes and QDs over time, it was demonstrated that the investigation of kinetics could effectively discern between different metals [48]. This research focused on the quenching kinetics induced by Fe^2+^ and Fe^3+^ ions on glutathione (GSH) capped-CdTe QDs. Both Fe^2+^ and Fe^3+^ can quench the fluorescence of GSH-CdTe QDs, but with different kinetics. Upon the addition of Fe^3+^, the fluorescence of GSH-CdTe QDs was rapidly quenched by approximately 18% within one minute, subsequently reaching equilibrium. Contrastingly, the addition of Fe^2+^ resulted in a gradual quenching of the fluorescence intensity of Cd-based QDs. The fluorescence decreased by approximately 65% within the first 5 min, followed by a slower decrease of 15% over the next 25 min. This distinct kinetic behavior suggests differences in the interaction between Fe^2+^ and Fe^3+^ with GSH-CdTe QDs (Figure 3a). Additionally, it was observed that different metals also exhibited distinct kinetic behaviors (Figure 3b). The observed variations in quenching behaviors among different metal ions for CdTe QDs can be attributed to differences in their electronic structures and dissimilar redox potentials. This understanding of the kinetics and mechanisms involved in the interaction between metal ions and CdTe QDs is crucial for the development of sensitive detection methods and the optimization of QD-based sensing platforms [48]. This work was pioneering in the utilization of the kinetic behavior of the interaction between QDs and target compounds. However, this approach was only applied to iron speciation in that study, without the use of chemometric models.

**Figure 4:**
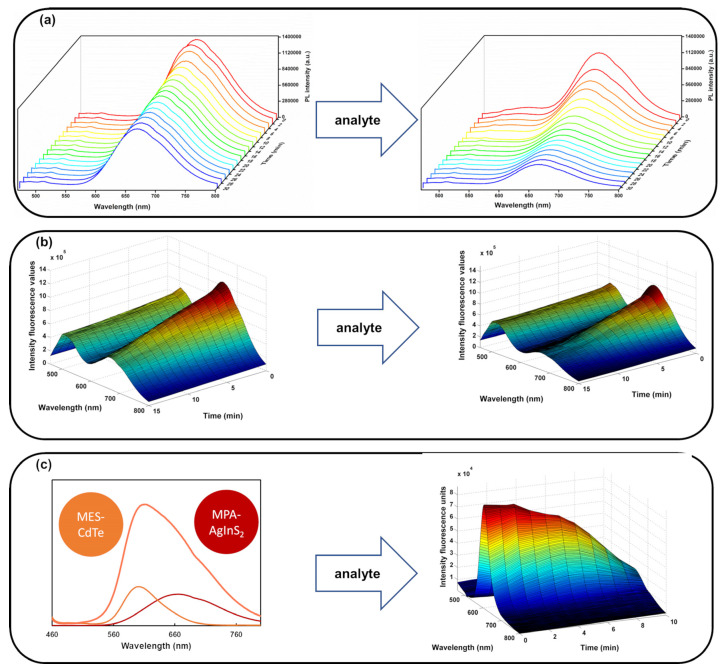
Alternative approaches for achieving effective kinetics-based sensing platforms using QDs: (**a**) nanoprobe with a single emitter for oxytetracycline detection; (**b**) multi-emission nanoprobe with distinct emission spectra in a ratiometric probe for the detection of histamine in foodstuffs; and (**c**) PL spectra of the nanohybrid probe composed of MES-CdTe/MPA-AgInS_2_ with the overlap of both individual nanoparticle emission bands by the determination of acetylsalicylic acid in pharmaceutical formulations. The colors represent the intensity of the emission, with red indicating higher intensity and blue indicating lower intensity.Adapted with permission from [37,39,50]. Copyright 2021 Elsevier, 2023 Elsevier, 2023 MDPI.

Choosing the optimal chemometric model and modulation strategy is crucial for ensuring the desired outcomes of the analysis when employing QDs as fluorescence probes. In this regard, various approaches have been investigated to develop a reliable sensing platform based on QDs:

(i) Using single-emitter QDs involves their interaction with the analyte, resulting in the modulation of their optical properties over time (Figure 4a). This interaction, which leads to various PL responses, is mainly influenced by the surface chemistry of the QDs as well as the stability of QDs upon the interaction with the target analytes. Indeed, the nanocrystal’s reactivity towards the analyte is determined by the distinct terminal functional groups of the capping ligand employed to stabilize the QD’s surface. Consequently, changes in optical properties can occur through alterations in PL intensity or shifts in the maximum emission wavelength.

(ii) Using multi-emitter probes involves combining various multi-colored fluorophores (including QDs and/or other molecules) with different affinities towards the analyte. This enables the gathering of complementary information, which is crucial for obtaining a distinctive profile of analyte responses. Indeed, through individual interactions with each single-emitter probe, targets can be more easily quantified and discriminated based on the cumulative nonspecific responses. This capability is achieved with multi-emitter nanoprobes designed for multipoint detection. The presence of multiple responses in the same probe significantly enhances the amount of available information, providing a more comprehensive and accurate understanding of the analyzed sample. In such multi-emitter probes, two fluorophores can be strategically used, obtaining an emission spectrum of the combined probe with well-separated emission bands, which allow for distinct signal detection and analysis (Figure 4b). Alternatively, the spectral overlap between the emission bands of the two fluorophores from the combined probe can be exploited (Figure 4c).

Abdollahi et al. [49] used three-dimensional kinetic data to determine copper ions. In the study, the authors investigated the modulation of the PL of L-cysteine (CYS)-capped CdS QDs in the presence of Cu^2+^ over time. The data were analyzed using various chemometric models, including multivariate curve resolution with alternating least squares (MCR-ALS) and partial least squares (PLS), after row-wise augmentation of the second-order data. MCR-ALS was employed to extract spectral profiles of all chemical species present, while PLS was used for quantifying the metal ions. Additionally, the authors examined the kinetic interaction of the nanoprobe with other ions (Ag^+^, Ni^2+^, and Hg^2+^), and they observed that each tested metal ion produced a distinctive emission spectra profile, indicating unique kinetic behaviors in the CdS QD PL. This observation suggests potential applications for accurately quantifying Cu^2+^ in the presence of interfering species and simultaneously determining co-existing metal ions that affect the QD PL.

**Figure 5:**
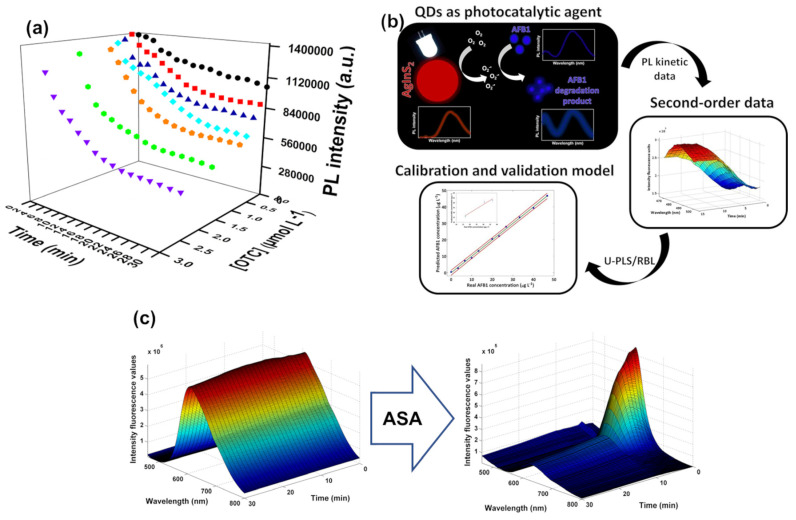
(**a**) Progression of the PL emission intensity of the AIS QDs at the maximum emission wavelength over time, both in the absence of OTC and upon the addition of increasing concentrations of OTC. (**b**) Schematic representation summarizing the detection principle of AFB1 via the photocatalytic process involving the mycotoxin and AIS QDs and AFB1 determination using the U-PLS model. (**c**) Second-order data PL spectra of the combined nanoprobe before and after the interaction with 35.6 mg L^−1^ of acetylsalicylic acid over 30 min. The colors represent the intensity of the emission, with red indicating higher intensity and blue indicating lower intensity. Adapted with permission from [37,39,59]. Copyright 2021 Elsevier, 2023 Elsevier, 2023 MDPI.

Our research group also developed a methodology using single-emitter QDs, thiomalic acid (TMA)—capped-AgInS_2_ (AIS) QDs. Initially, a ratiometric sensing platform comprising two dynamic fluorophores was developed for analytical purposes. In this platform, oxytetracycline (OTC) served as both the analyte and fluorophore, while the QDs dynamically responded to the presence of the target molecule. Consequently, as the concentration of OTC increased, the corresponding emission band also increased, while the emission of AIS QDs was gradually suppressed. The proposed ratiometric sensing approach was tested for determining OTC in a commercially available veterinary pharmaceutical formulation. The results showed that the excipient, lactose monohydrate (94.5% of the sample content), significantly influenced the ratiometric measurements. Although the inhibition of AIS QDs’ PL emission remained unaffected, the presence of the excipient hindered the emergence of the OTC emission band. Due to this excipient effect, the feasibility of using the proposed ratiometric sensing approach for OTC determination in pharmaceutical formulations was impracticable. To circumvent the presence of the excipient in pharmaceutical formulations, the kinetic behavior of AIS QDs’ PL quenching in the presence of OTC was evaluated. Immediately after the addition of OTC (0 min), a slight quenching of the PL of AIS QDs was observed, which intensified with increasing antibiotic concentration over time. This gradual enhancement of the quenching effect on QDs’ PL emission with reaction time emphasized the potential of this kinetic approach for OTC determination (Figure 5a). The acquisition of kinetic data not only reduced the limit of detection (LOD) but also enhanced sensitivity and selectivity. Therefore, the corresponding kinetic data were analyzed using unfolded partial least squares (U-PLS) to overcome selectivity issues. This was achieved through the acquisition of second-order data, which proved beneficial for quantifying OTC in the presence of uncalibrated lactose monohydrate.

Despite their effective use as PL sensors, QDs also exhibit excellent photocatalytic properties [6,51]. Effectively, their ability to generate reactive oxygen species (ROS) in aqueous media upon irradiation with ultraviolet (UV) and visible light has been exploited for several applications. This photocatalytic capacity has led to diverse applications in environmental remediation [52,53,54], microbiological interventions [55,56], and analytical chemistry [7,57,58]. Notably, QDs have been utilized to eliminate persistent pollutants in aquatic environments, serve as antimicrobial nanomaterials, and facilitate the determination of analytes, namely, analytes with antioxidant properties.

In another work, the ability of Cd-free AIS ternary QDs to generate ROS, upon visible light irradiation, was used for Aflatoxin B1 (AFB1) determination [59]. The photogenerated ROS induced the formation of highly fluorescent AFB1 degradation products, which were used for mycotoxin monitoring. This work demonstrated that although semiconductor QDs were not suitable for direct use as PL sensing platforms for AFB1 determination, AIS QDs showed potential as a photocatalytic agent, enabling mycotoxin detection. The gradual increase in PL emission (more accentuated in the first 15 min) can be attributed to the increased fluorescence of the photodegradation products compared to the native fluorescence of AFB1. Effectively, the photodegradation products resulting from the photocatalytic activity of QDs increased in solution over time (Figure 5b). In contrast, the fluorescence of the AFB1 solution showed minimal change without QDs, showing the central role of QDs as photocatalytic agents. Subsequently, the use of a flexible multivariate calibration model like unfolded partial least squares followed by residual bilinearization procedure (U-PLS/RBL) allowed for the extraction of useful information from the PL dataset, making the accurate determination of AFB1 in some foodstuffs possible, even in the presence of unknown interfering species not included in the calibration set [59].

The use of nanoprobes encompassing multiple emitters with multipoint detection, combined with the analysis of PL data using appropriate chemometric models, presents a viable strategy for quantifying analytes in complicated samples. This approach provides complementary and valuable information, allowing for an analytical methodology with enhanced accuracy and selectivity. Among the different types of multi-emitter nanoprobes, ratiometric sensing platforms, involving the combination of QDs of different sizes or nature, each emitting light at specific wavelengths, have been most commonly explored. Our research group combined CDs (non-reactive reference fluorophores) and 3-mercaptopropionic acid (MPA)-capped AIS QDs (reactive fluorophores whose PL intensity is modulated by the target analyte) to determine histamine in foodstuffs [50]. In this study, two different ratios of AIS QDs and CDs were explored (different CD dilutions) to assess the effectiveness of the multi-emitter ratiometric sensing platform in detecting histamine. The results indicated that the ratiometric probe’s performance varied in terms of the determination coefficient (R_2_C) and root mean square error of calibration (RMSEC) for different combination ratios. This demonstrates that the selection of the sensing platform is essential, even in cases where the fluorophore is inert to the presence of the target analyte. Moreover, the assessment of two chemometric models, specifically N-way partial least squares (N-PLS) and U-PLS, involves evaluating them using validation samples across different time intervals. This evaluation occurred within the first 15 min. Although both chemometric models proved capable of accurately determining histamine in complex matrix samples, the N-PLS model outperformed the U-PLS model in terms of prediction accuracy. Additionally, accurate results were attained with just 5 min of kinetic spectral acquisition. Therefore, by making a better compromise between time consumed per sample and the results’ accuracy, the shorter time was selected, thus allowing an increase in the sampling rate. The developed methodology demonstrated accuracy, reliability, and simplicity, and at the same time, it was less labour-intensive and more environmentally friendly compared to the reference procedures, namely high-performance liquid chromatography or capillary electrochromatography. Effectively, utilizing nanoprobes with multiple emitters and multipoint detection, along with employing suitable chemometric models to analyze PL data, provides a practical method for quantifying analytes in complex samples such as tuna, tomato, and hake fish [50].

Lastly, an interesting approach was developed using a dual-emitter fluorescent probe that combines binary CdTe and ternary AIS QDs for the determination of acetylsalicylic acid (ASA) [39]. Matrices were acquired using a time-based approach (kinetic method) to ensure the gathering of second-order data capable of exploiting the second-order advantage. Contrarily to the abovementioned work, the PL spectrum of the multi-emitter nanoprobe displayed a significantly broad band, with undefined maximum emission wavelengths. This was attributed to the overlap of the individual nanoparticle emission bands, and this overlapping phenomenon led to an enhancement in the PL intensity of both emission bands. The presence of ASA caused a gradual PL quenching of the combined nanoprobe, which was more pronounced in the first 10 min of the interaction (Figure 5c). Additionally, four distinct chemometric models, namely U-PLS, N-PLS, multilayer feed-forward neural networks (MLF-NNs), and radial basis function neural networks (RBF-NNs), were evaluated to determine the most suitable chemometric approach. While some minor differences were observed during calibration, the overall accuracy did not significantly differ between the models tested. The best results were obtained when fluorescence signals were mean-centered. In the validation phase, all models demonstrated high accuracy, confirming the reliability of the proposed methodology for ASA quantification in pharmaceutical samples [39].

This study conducted an important investigation to evaluate the stability of various synthesized QDs. Effectively, the stability of QDs is of paramount importance in kinetic assays since ensuring the stability of QDs guarantees consistent and reliable performance throughout the assay, minimizing fluctuations in signal intensity or emission spectra. Unfortunately, some QDs exhibited a significant inhibition of PL emission (~70%) within the first 15 min [39]. The predominant method for optimizing synthetic routes for QDs remains univariate approaches. Furthermore, this synthesis is generally optimized considering the maximum emission wavelength or their quantum yield, almost always neglecting their stability. Since the stability of nanomaterials is vital for implementing kinetic assays, this parameter must receive greater consideration in future research.

**Table 1:** Analytical approaches exploring the use of kinetic data using QDs as a sensing platform.

Analytes	Sensing Platform	Chemometric Tool	Time Acquisition	LOD	Ref.
Fe^2+^ and Fe^3+^	GSH-CdTe	n.a.	30 min.	5 nmol L^−1^	[48]
Cu^2+^	CYS-CdS	MCR-ALS PLS	30 min.	13 nmol L^−1^	[49]
OTC	TMA-AIS	U-PLS	30 min.	0.144 μmol L^−1^	[37]
AFB1	MPA-AIS	U-PLS	15 min.	1.2 µg L^−1^	[59]
Histamine	CDs@MPA-AIS	U-PLS N-PLS	5 min.	1.26 mg L^−1^	[50]
ASA	MES-CdTe@MPA-AIS	U-PLS N-PLS MLF-NNs RBF-NNs	10 min.	2.82 mg L^−1^ 3.10 mg L^−1^ 3.38 mg L^−1^ 3.26 mg L^−1^	[39]

n.a. means not applicable.

## 3. Conclusions and Prospects

Using kinetic data in QD-based PL sensing platforms is a promising strategy for improving the results obtained in the discrimination and quantification of analytes, which is crucial in analytical chemistry. The accurate quantification of the concentration of the analytes, even at minimal levels and in the presence of interfering species, is important for multiple applications, such as environmental monitoring and biomedical diagnostics. Kinetic approaches can provide comprehensive knowledge about complex systems by collecting a higher amount of data, which allows, for example, the quantification of analytes in the presence of uncalibrated species.

Additionally, obtaining kinetic data is accessible since routine laboratory instrumentation is available. Employing kinetic assays provides an effective solution for circumventing interference issues that commonly occur in complex sample matrices. Using second-order data with appropriate chemometric analysis allows for obtaining second-order advantage, which enables the accurate quantification of analytes, even in the presence of uncalibrated interferents. Therefore, this approach can successfully distinguish analytes from interfering and uncalibrated substances, especially in samples with unknown and/or variable compositions, such as environmental, food, or biological samples. This ability is particularly important for the application of QDs in real-world samples. Unfortunately, only a small number of scientific studies have explored the use of kinetic data employing QDs as PL emission nanoplatforms.

Moreover, kinetic analysis using quantum dots as PL probes can benefit from developments in other scientific areas. An example is an improvement in nanomaterial stability, which, in several cases, loses its stability in a few hours, impairing the application of kinetic measurements. In this sense, the development of synthesis strategies should prioritize the stability of QDs to foster the use of this kinetic approach in various analytical applications. Furthermore, silica coating has been explored as a strategy to enhance the stability of QDs, with several reported applications in some areas, namely, chemical analysis and biocatalysis. However, coating the surface of QDs with silica can compromise the QD’s reactivity, making them less prone to establishing chemical interactions with the target analyte, which may be a significant limitation depending on the intended analytical application. Indeed, more studies may be performed to obtain the best compromise between the stability and reactivity of the nanoparticles. Additionally, the increased interest in synthesizing eco-friendly alternatives for conventional Cd-based QDs is expected to contribute to the development of greener analytical methodologies with minimal ecological impact. These nanoparticles should provide similar performance in terms of sensitivity for kinetic analysis.

Another important improvement in kinetic methodologies is their portability, which ensures a broader accessibility. A promising alternative is the use of smartphone-based analytical platforms, employing the capabilities of mobile devices for kinetic data collection and analysis. As smartphones are used extensively in our daily lives, researchers can enable their worldwide use to perform advanced kinetic analyses more simply. Visual detection techniques allow for the identification and/or quantification of analytes without requiring complex external instruments, using color changes detected during interactions with PL QDs. Although QD-based visual sensing methods allow for qualitative or semi-quantitative evaluations through visual inspection, the use of smartphones presents an interesting solution for immediate and on-site quantitative measurements without depending on complex laboratory equipment. High-quality imaging capabilities available in most modern smartphones, combined with proper software applications that can convert the acquired color data into RGB values, emerge as efficient sensing platforms for monitoring various target species. To the best of our knowledge, all the studies that use smartphones for the capture of RGB data focus on single-point measurements. However, continuous advancements in smartphone camera resolution and image processing capabilities indicate their potential application in kinetic measurements in the future. Exploring this potential could open new perspectives for real-time portable kinetic analyses using smartphone-based platforms.

As final remarks, the use of kinetic data for QD-based analytical nanoplatforms promises to foster the development of analytical methodologies through increased sensitivity, which will improve the discrimination capacity, as well as the accurate quantification of analytes in the presence of uncalibrated interfering species. These features are crucial for providing comprehensive knowledge about complex systems and developing reliable analytical techniques.

## Data Availability

The raw data supporting the conclusions of this article will be made available by the authors upon request.

## Abbreviations

The following abbreviations are used in this manuscript:

AFB1	Aflatoxin B1
AIS	AgInS2
ASA	acetylsalicylic acid
CDs	carbon dots
CYS	L-cysteine
EEM	Excitation–Emission Matrix
FRET	Förster Resonance Energy Transfer
GSH	glutathione
LEDs	light-emitting diodes
LOD	limit of detection
MCR-ALS	multivariate curve resolution with alternating least squares
MIPs	molecularly imprinted polymers
MLF-NNs	multilayer feed-forward neural networks
MPA	3-mercaptopropionic acid
N-PLS	N-way partial least squares
OTC	oxytetracycline
PL	photoluminescent
PLS	partial least squares
QDs	quantum dots
QY	quantum yield
R2C	determination coefficient
RBF-NNs	radial basis function neural networks
RGB	red, green, and blue
RMSEC	root mean square error of calibration
ROS	reactive oxygen species
TMA	thiomalic acid
U-PLS	unfolded partial least squares
U-PLS/RBL	unfolded partial least squares/residual bilinearization
UV	ultraviolet
